# Vapor Sensing with Polymer Coated Straight Optical Fiber Microtapers Based on Index Sensitive Interference Spectroscopy of Surface Stress Birefringence

**DOI:** 10.3390/s20092675

**Published:** 2020-05-08

**Authors:** Alexandra Blank, Gabriel Guendelman, Yoav Linzon

**Affiliations:** 1 School of Mechanical Engineering, Faculty of Engineering, Tel Aviv University, Tel Aviv 69978, Israel; 2 Chemical Physics Department, Weizmann Institute of Science, Rehovot 76100, Israel; gabriel.guendelman@weizmann.ac.il

**Keywords:** optical resonators, optical sensing, microstructured fibers

## Abstract

Optical microfiber tapers provide an advantageous platform for sensing in aqueous and gas environments. We study experimentally the photonic transmission in optical fiber tapers coated with polymethyl methacrylate (PMMA), a polymeric material widely used in optical applications. We demonstrate a durable and simple humidity sensing approach incorporating tapered microfibers attached to silicon (Si) substrate coated with active polymer layer. A model is described for the load stress effect on the birefringence giving rise to interferences in the transmission spectra, strongly dependent on the coating layer thickness, and disappearing following its slow uniform removal. The sensing approach is based on characterization of the interference patterns observed in the transmission spectra of the taper in the NIR range. The device demonstrated persistent detection capability in humid environment and a linear response followed by saturation to calibration analytes. In each analyte of interest, we define principal components and observe unique calibration plot regimes in the principal component space, demonstrating vapor sensing using polymer coated microtapers.

## 1. Introduction

Sensing using optical methods has been an active field of research in recent years [1,2,3], due to the inherently superior capabilities offered by complex photonic structures [4,5]. Specifically, microstructures incorporated within optical fiber tapers transmitting near-infrared (NIR) light have found comprehensive applications in a variety of sensing scenarios, including in liquid [6,7,8,9], gas [10,11], and other physical [12,13,14,15,16] and chemical [17,18] media. In particular, the chemically inert nature of silica fiber [19] and Si substrates [20] makes their combination a leading platform for integration with reactive layers for controllable chemically-sensitive response tailored to vapor specificity [21,22], and measuring additional quantities such as temperature together with the vapor content [23]. Specificity, together with good resolution, usability over many cycles, and low power consumption, remain key goals for future vapor sensors to be deployed in large scale applications.

In the specific context of optical fiber tapers with light transmitted in the NIR, bare microknot resonators (MKRs) have been shown to operate in closed humidity chambers with a linear resonance wavelength shift of up to 6.15 pm per 1 percent of water relative humidity (RH) increment [24]. Rather than giving wavelength shifts alone, a useful quantity is the relative phase shift (PS), where the relative resonance shift $\Delta \lambda =\lambda \left(RH\right)-\lambda_{0}$ is normalized by the baseline central wavelength $\lambda_{0}$, providing the unitless figure-of-merit for the sensor, PS = $\frac{\Delta \lambda }{\lambda_{0}}$. With the central wavelength corresponding to the typical telecom-applicable NIR input wavelength (≈1550 nm), in [24] the maximum PS value achieved was PS ≈ 0.004, or 4 ppt at saturated atmosphere (RH = 100%). Stress and strain detection using silica MKRs have also been recently demonstrated [25]. Polymer optical fibers, typically produced from polymethyl methacrylate (PMMA), were recently presented as an advantageous platform for curvature, force, strain and stress sensing applications offering flexibility, compactness, and lightweight [26,27,28,29]. Passive devices typically rely on condensation near the substrate or point stresses giving rise to resonance shifts in folded microtapers. In this paper, we propose and demonstrate that in straight rather than folded microtapers, uniformly coated with active polymer layer, the swelling of the top layer exposed to the ambient vapor as a function of humidity in the chamber gives rise to interference patterns attributed to surface stress birefringence effect, with significant phase shifts linear with RH below saturation, and parameters unique to different volatiles used.

## 2. Model

**Figure 1:**
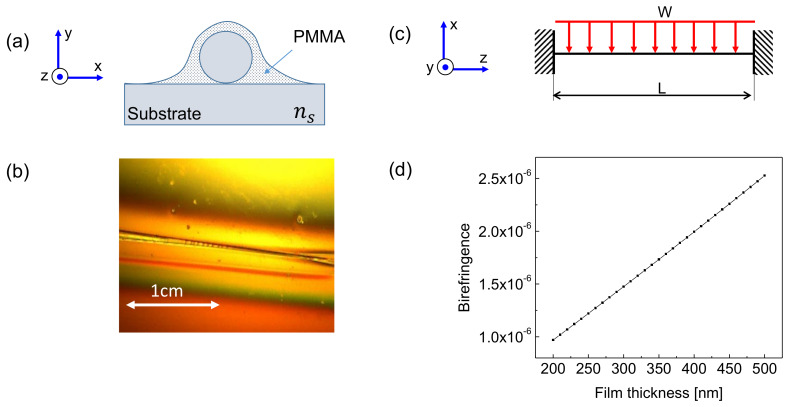
(**a**) Schematics of the cross-section and axes definition in a coated cylindrical taper on substrate; (**b**) microscope image of the coated taper as viewed from above. (**c**) Schematics of the side view in doubly-clamped fiber segment with the mechanical model of load distribution; (**d**) birefringence strength as a function of the film thickness as calculated using the model (Equations (1)–(4)).

The problem of stress-induced birefringence in optical fibers of circular cross-section has been broadly studied [12,30]. However, in the case of tapered fibers, where the waist diameter is in the microscale, the effect of stress-induced birefringence can become more pronounced dependent on the local pressure introduced. Figure 1a shows the cross-section of the unstressed polymer coated taper on top of the Si substrate, and Figure 1b illustrates a microscope image of a coated tapered viewed from above (low magnification). Figure 1c depicts the side view of the fiber segment, assumed to be doubly-clamped and with the mechanical model of weight load distribution. We will concentrate here on coating thicknesses that can easily be obtained with good surface uniformity the using standard procedure of spin coating, namely up to 450 nm thickness in PMMA deposition.

It is well known that stress-induced changes along the principal direction in an initially isotropic cylindrical fiber can be described by the following set of equations [31]:

(1) $$\begin{array}{c} n_{x}=n_{0}+C_{1}\sigma_{x}+C_{2}\left(\sigma_{y}+\sigma_{z}\right),\\ n_{y}=n_{0}+C_{1}\sigma_{y}+C_{2}\left(\sigma_{x}+\sigma_{z}\right),\\ n_{z}=n_{0}+C_{1}\sigma_{z}+C_{2}\left(\sigma_{x}+\sigma_{y}\right), \end{array}$$

where in Cartesian coordinates $C_{1}$ and $C_{2}$ are the stress-optic coefficients of the fused silica fiber; $n_{0}$ is the isotropic refractive index of the relaxed fiber material; $n_{x}$, $n_{y}$, $n_{z}$ are the stress-induced refractive indices along the principal axes; and $\sigma_{x}$, $\sigma_{y}$, $\sigma_{z}$ are the stress components along the principal axes *x*, *y*, and *z*, respectively. Assuming that *z* is the principal optical propagation direction (see Figure 1a,b) and solving for the difference between the axial and transverse refractive indices, for stress-induced birefringence strength we obtain:

(2) $$n_{z}-n_{x}=\Delta n=C\left(\sigma_{z}-\sigma_{x}\right),$$

where $C=C_{1}-C_{2}$ is the stress-optical coefficient difference.

Following the terminology used in [30], we consider here the case of extrinsic birefringence caused by the stress induced by the weight distribution of the polymer coating. We note that upon swelling of the active layer its’ effective mass is increased linearly with the swelling, until saturation. Equation (2) represents the stress-optical effect relating the difference in the taper refractive indices between longitudinal and transverse directions to the residual stress. Assuming a simple mechanical model shown in the Figure 1b, where the taper above the substrate is considered as a cylindrical beam fixed at both its sides and subjected to a uniformly distributed load along its length, we analyze the stress distribution along the taper profile. Given that the PMMA coating is distributed uniformly both in dry (unswelled) and wet conditions, the local taper cross-section stress as a function of the longitudinal distance *z* along the taper is given by [32]:

(3) $$d\sigma \left(z\right)=\frac{WL}{2Z}\left(\frac{1}{6}-\frac{z}{L}-{\left(\frac{z}{L}\right)}^{2}\right),$$

where *W* is the total load on the taper, Z=I/d is the section modulus of the taper cross-section, *d*, according to the section modulus definition given in [32], is the distance in the section plane between the taper neutral axis and its extreme edge, and is equal to the taper cross section radius in the particular case of a circular cross-section, *I* is the taper cross-section moment of inertia, *z* is the distance along the taper longitudinal axis where the stress is calculated, and *L* is the total length of the loaded section (0<z<L). The assumption of a homogeneous coating layer is based on the spin-coating process typically used in device fabrication and intended for uniform thin film deposition on flat surfaces. The film thickness and its uniformity is measured using film thickness probe tabletop reflectometer connected to a microscope station. Following Equation (3) and given that the light propagation is through the whole fiber length, and thus the transmission being sensitive to the accumulated effect, the total stress can be estimated as an integral over the whole segment:

(4) $$\sigma_{z}=\int_{0}^{L}d\sigma \left(z\right).$$

Substituting Equation (3) in Equation (4), integrating, and plugging into Equation (2), we can estimate the total birefringence caused by the stress in the taper induced by PMMA coating swelling, assuming no additional axial stresses in the untensioned taper exist ($\sigma_{x}=0$). Using the parameters: taper waist diameter 7 μm; taper waist length L=7.62 cm; coating of PMMA A4 of mass density 1.18 ($g/cm^{3}$); stress-optic coefficient $C=3.184\times 10^{-12}$; maximum polymer film thickness t=450 nm, we obtain for stress-induced birefringence the value of $\Delta n=2.3\times 10^{-6}$ (Figure 1d).

With the application of vapor sensing in mind, the estimation of stress-induced birefringence in the taper is of importance due to the swelling of the polymer film with ambient humidity, increasing the load *W* with added vapor content. Figure 1d shows the birefringence caused by the weight of the coating layer as a function of the film thickness. The values of the film thicknesses shown in the figure correspond to those typically attainable during the standard spin-coating procedure (200−450) nm, and the subsequent values achieved for linear RI variations are in the range (1−2.5)$\times 10^{-6}$.

The subsequent propagation of light through the fiber having both ordinary $n_{x}$ and extraordinary $n_{z}=n_{x}+\Delta n$ indices gives rise to the superposition of light with both frequencies $\omega_{x}=kc/n_{x}$ and $\omega_{z}=kc/n_{z}$, where *c* is the speed of light and k=2π/λ is the wave number, λ being the free-space wavelength. In the superposition of both modes, observed in the transmission signal, a modulated envelope with a low frequency spacing of $\Delta \omega =\omega_{x}-\omega_{z}$ will be present on top of the high-frequency optical waves. This modulation corresponds to interference pattern observed in the transmission spectra. Estimating the maximum visible interference spacing, for NIR light in the range around λ = 1.5 μm (central frequency $\nu_{0}$ = 200 THz), using the above model results, we obtain: $\Delta \nu =\Delta \omega /2\pi =\frac{c}{\lambda }\frac{\Delta n}{n_{x}n_{z}}\approx 200$ GHz, corresponding in terms of the wavelength to a period of $\Delta \lambda =\frac{c}{\nu_{0}^{2}}\Delta \nu$ = 1.5 nm, which can be well observed with standard NIR optical spectrum analyzer (sweeping tunable IR laser and detector pair), as detailed in the following experimental sections. We emphasize that here the observed effect arises from index-sensitive interference patterns induced by surface stress birefringence in a straight taper, rather than knot or loop resonances previously observed in circular tapers. In coated MKRs or MLRs, we estimate that a combination of the spectral effects (both resonances and stress-induced interferences) will simultaneously be observed.

## 3. Device and Experiment Details

**Figure 2:**
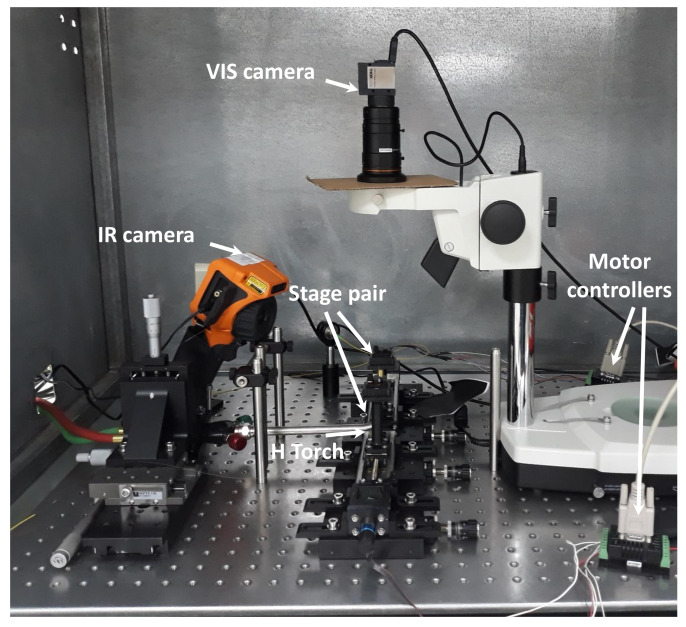
Pulling station including a pair of long-travel motorized stages with clamped posts, hydrogen torch producing a hot flame below the taper, and a pair of visible (above) and IR (side) control cameras used during microtaper preparation starting from a standard single-mode fiber.

Figure 2 illustrates a photograph of the pulling machine incorporating a hydrogen gas generator creating up to 120 sccm flow, two precisely aligned stepper motors and a torch used as a heat source. The setup is equipped with an IR camera to control the flame profile and instantaneous temperature, while the top view of the taper shape is observed using a stadard CCD camera at × 4 magnification, with both cameras connected to the LabView application monitoring their parameters in real-time during the tapering process. Following the procedure of [33], the standard SMF is subjected to buffer coating removal and cleaning, and then it is placed in the setup connected to a power meter monitoring its NIR transmission during pulling and subsequent thinning.

After achieving the desired taper waist diameter, the taper is placed on top of a Si substrate and subjected to spin-coating resulting with a thin layer of polymethyl methacrylate (PMMA, diluted in 4–11% Anisole). The polymer layer is spinned using a commercial desktop precision spin coating system P-6708D, followed by 1 min bake on a hot plate at 180 ${}^{\circ }$C. The thickness of the polymer film is measured using film thickness probe tabletop reflectometer FTP-Advanced connected a microscope station. The thicknesses of the deposited polymer films achieved (Figure 1a) vary between 200–950 nm, in correspondence with spin speed curves available in PMMA datasheet.

**Figure 3:**
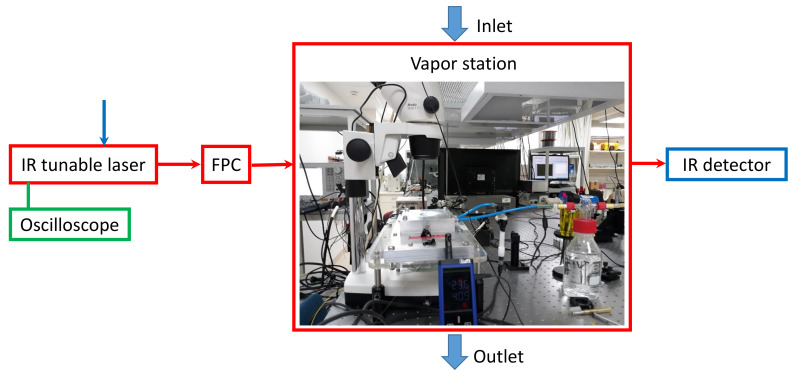
Schematics of the experimental setup including vapor measurement station: FPC–input light fiber polarization controller. Arrows above and below are showing the inlet and outlet of the vapor gas mixed in N${}_{2}$.

Figure 3 shows the schematics of the experimental setup including the vapor measurement station. The device is placed inside the humidity chamber and coupled to the IR laser (tunable in the range 1.5–1.6 μm) through the polarization controller. The transmitted light output is connected to the built-in IR detector with a resolution 0.1 nm. To introduce the analyte vapor in the chamber we use a bubbler bottle with its inlet connected to a dry nitrogen line and its outlet connected to the vapor chamber mixed with analyte vapor. The humidity sensing properties of the device are investigated at room temperature, where analyte vapors used were deionized water, ethanol, and methanol, respectively, all 95% absolute. The humidity and temperature in the chamber were measured using Lufft XC200 handheld sensor and its provided software for data acquisition. The humidity in the XC200 is set to be measured as relative (in percent) rather than absolute, with the vapor pressure of each analyte used in each case. During the experiment we monitored the transmission spectrum through the device along with a continuous measurement of the temperature stability and humidity level. Optionally the setup in Figure 3 can incorporate an oscilloscope for real-time transmission measurements, rather than point-by-point wavelength sweeping. We use single-mode tapered optical fibers coated with thin layers of PMMA polymer deposited on flat 3-inch Si substrates. Tapers of high uniformity (5–7 μm in waist diameter) were produced starting from standard single-mode low-loss fused silica fibers (Thorlabs SMF; wavelengths of operation 1.5–1.6 μm) using a custom-made tapering machine implementing the flame-brushing pulling procedures described in [33]. Herein we consider the parameters of the fiber prior to tapering as 125 μm for a clad diameter and 9 μm for a core diameter, respectively.

## 4. Results and Discussion

**Figure 4:**
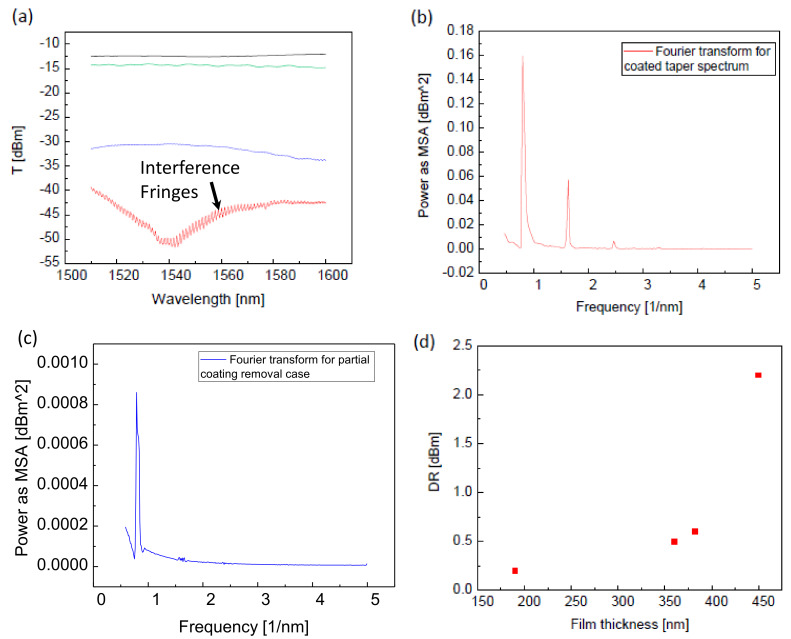
(**a**): IR transmission spectra of the uncoated taper (black line), fully coated taper (red line), and after slow coating removal in Acetone vapor exposure: blue line—after 2 h, green line—after 2 days. (**b**): Fourier transform of the fully coated taper spectrum. (**c**): Fourier transform of the spectrum after first coating removal. FFT spectra are given in Mean Square Amplitude (MSA) Power as a function of spatial frequency in (1/nm). (**d**): Dynamical range of the patterns as a function of the film thickness.

To test the thickness dependent properties of the coated taper, as well as its cleaning procedure, we performed partial coating removal experiment. The PMMA coated taper was placed inside the chamber saturated with Acetone vapor. During purging the chamber inlet had continuous flow from the bubbler bottle filled with liquid Acetone subject to high flow of N${}_{2}$ gas. After each exposure to Acetone vapor, the coating thickness was measured. We have monitored the changes in the transmission spectrum of the device in terms of its baseline and interference patterns that have become smaller in dynamical range after coating thinning. Figure 4a illustrates the transmission spectra of the uncoated taper (black line), coated taper (red line, 450 nm coating), taper after 2 h in the Acetone chamber (blue line, 382 nm coating); taper after 2 days of subsequently being subjected to the Acetone vapor (green line, 190 nm coating). As evident in Figure 4a, the uncoated tapers demonstrate high transmission, relatively constant spectrum in the range 1500–1600 nm with no interference patterns (black line in Figure 4a). Following coating, the tapers demonstrate a pronounced interference pattern with dynamical range of up to 2 dBm (red line in Figure 4a). Figure 4b shows the Fourier transform of the coated taper broad spectrum corresponding to the red line of Figure 4a. The transformed signal in Figure 4b reveals the two dominant frequencies in the transmission spectrum of the coated taper being 0.8/nm (corresponding to wavelength period of 1.25 nm, similar to the estimation of the model) and 1.6/nm corresponding to the second harmonic (wavelength period of 0.62 nm). In the Fourier transform corresponding to partially removed coating (Figure 4c) only the first harmonic is observed at frequency 0.95/nm (wavelength period 1.05 nm). Thus, the thickness reduction of 15% resulted in interference wavelength decrease of 16%, which is linear up to experimental uncertainties, as predicted by the model. During the coating removal test we also observed revival of the transmission resulting from exposure to Acetone vapor, yielding revival from −45 dBm to −30 dBm after two hours (blue line in Figure 4a), and to −15 dBm following two days in Acetone exposure, almost within the initial uncoated level of −12 dBm (green and black lines in Figure 4a, respectively). Along with the total transmission revival, the reduction of the interference pattern visibility was observed. Barely any interference patterns were visible in the device transmission spectrum after two days of the Acetone vapor exposure. Figure 4d shows the dynamical range (visibility) of the interference patterns as a function of polymer film thickness, clearly showing the diminishing of the interference pattern with coating thinning, hinting at stress relief on the taper section buried in the coating. Table 1 summarizes the experimentally estimated DR values of the interference patterns at different PMMA film thicknesses.

**Figure 5:**
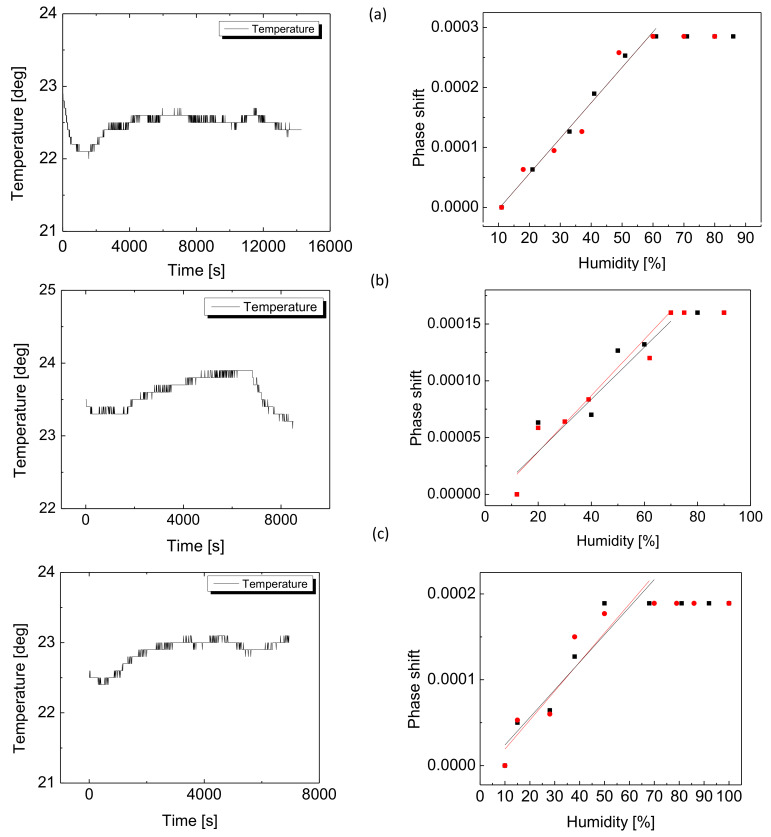
Thick 450 nm coated straight taper results: temperature stability (left) and relative phase shifts as a function of relative humidity (RH) levels, in exposure to: (**a**) DI water, (**b**) Ethanol, (**c**) Methanol.

Figure 5 shows the 450 nm coated device behavior in controlled humid environments. We tested the humidity sensing properties of the device using three analyte vapors mixed in dry N${}_{2}$ gas: deionized (DI) water (Figure 5a), Ethanol (Figure 5b) and Methanol (Figure 5c). During the experiment we monitored the humidity and temperature in the vapor chamber. All the left panels in Figure 5 show the temperature as a function of time measured using a commercial Lufft XC200 sensor having the resolution 0.1 ${}^{\circ }$C and indicate relative stability of the room temperature during the whole experiment, each one lasting between 2 and 3.5 h. We monitored the transmission spectra of the device in steps of 10% with humidity, each time reaching stabilized RH level within the chamber, starting from a dry chamber in nitrogen flow and slowly introducing the humid flow with increasing levels of bubbling flow rate. The Lufft sensor is also used for relative humidity measurements and has the resolution 0.1% RH. The limiting factor in the time response was stabilization of the vaporized humidity in the chamber of volume 2 liters, which was 5–8 min in intervals of 10% RH increase or decrease. Instantaneously, however, during the stabilization interval, the response of both the calibration and fiber sensors is estimated to be significantly faster, of the order of a few seconds. In all the experiments the same device was used. We note that after each humidification experiment the sensor was dried by introducing high-flow dry nitrogen in the chamber. We found that in humid environment, the transmission spectrum is phase shifted with respect to the spectrum of the same device at the lowest humidity setting. The linear phase shift is attributed to the index change of all refractive indices of the coating adsorbed with analyte, up to its saturation. The phase shifts (PS) have been experimentally derived from the transmission spectra, with the lowest humidity level (dry nitrogen) as the reference. We tracked PS in both humidification (black lines in Figure 5) and re-drying (red lines in Figure 5) stages and observed full linearity in the results. Black squares in Figure 5 correspond to the humidification stage, whereas red circles correspond to the re-drying phase showing full reversibility without hysteresis. In all analytes we observed the linear response of the sensor, with the saturation levels slightly different in each particular analyte. As observed from the Figure 5 (right panels), in DI water saturation starts at 60% humidity, in Ethanol at 70%, and in Methanol at approximately 50%. The total PS levels obtained were also different and particular to analytes, in the range from 1.5×10${}^{-4}$ in Ethanol to 3×10${}^{-3}$ in DI water. For the polymer-coated taper to serve as an efficient vapor sensing device we can estimate its detection capabilities in terms of sensitivity and range, using spectral response of the device in PS-humidity space. Thus, the sensitivities to the calibrated analytes can be estimated as slopes of the linear functions Δ(PS)/Δ(RH) and the range of the sensor can be determined from the saturation level particular to each analyte. Below we compare our sensor with other optical methods for vapor sensing described in recent works. The figures of merit, namely the sensitivity and range, given for water vapor, are summarized in Table 2.

**Figure 6:**
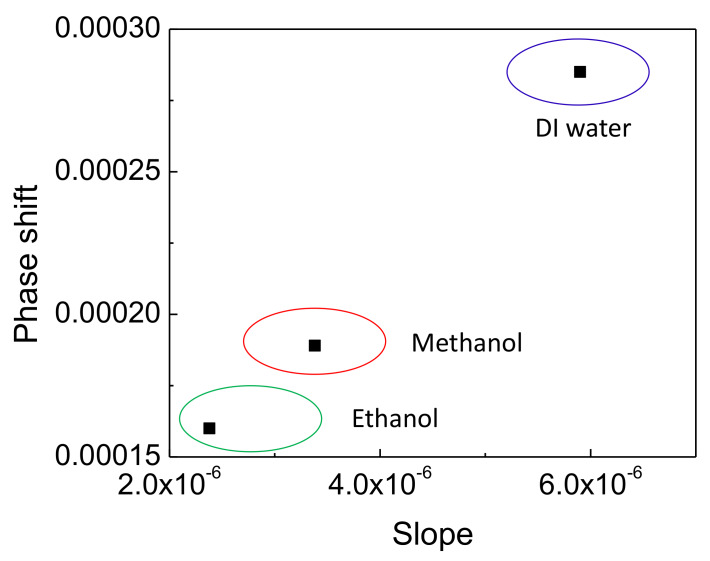
Analyte calibration plot in principal component space of phase shift versus slope.

In the interest of calibration of ambient vapors we defined the 2D analyte calibration plot for DI water, ethanol and methanol, where the total phase shifts and slopes of the sensor response in Figure 5 were chosen as principal components (Figure 6). Data points corresponding to each singular volatile organic compound analyte are enclosed by ellipses for clarity. It was found that the PS versus slope of all analytes were on nearly a line in the 2D space. In favor of recognition derived from the calibration curves, specifically with the three calibrated volatile compounds, we note that one could use RH levels lower than full saturation, estimating the local slope at small RH changes and comparison with the calibration plot, thanks to the good separation between the values of the horizontal principal component. In order to compare the vertical principal component, one would require to reach saturation and measure the total phase shift.

## 5. Conclusions

In this paper we presented a simple and durable humidity sensing approach incorporating tapered optical fibers on Si substrate and coated with an active polymer layer. We show theoretically that the transmission spectrum of coated tapers possess interference patterns induced by the stress applied to the taper due to the coating weight load. The interference patterns are strongly dependent on thickness of the coating layer and disappear in upon coating removal. The sensing approach is based on characterization of the interference patterns observed in the transmission spectrum of the optical taper in the NIR wavelength range. The fully coated device demonstrated persistent detection capability in humid environment and linear response to calibrated analytes. Each volatile organic analyte tested (DI water, ethanol, and methanol) defined a unique calibration area in principal components space. This demonstrates that polymer-coated single-mode optical microtapers, operating in a humid environment, are viable in humidity sensing applications based on index-sensitive interference spectroscopy of surface stress birefringence.

## Figures and Tables

**Table 1:** Experimentally estimated dynamical range values in polymer films of different thicknesses.

Film Thickness [nm]	DR of the Patterns [dBm]
450	2.2
382	0.6
360	0.5
190	0.2

**Table 2:** Comparison of the polymer coated straight microtaper-based sensor reported in this work with other sensors recently reported, in sensitivity to relative humidity (RH) percent change and total range of sensitivity.

Reference	Sensitivity	Range of Sensitivity [%]
This work	6×10${}^{-6}$/%	10–60
[24]	5.95 pm/%	30–94
[34]	44.2 pm/%	60–98.5
[35]	0.06 pm/%	15–85
[36]	9.57×10${}^{-5}$	5–97
[22]	0.51 nm/%	10–90
[23]	1.0072/%	35–71

## Abbreviations

The following abbreviations have been used in this paper:

SMF	Single mode fiber
Si	Silicon
PMMA	Polymethyl methacrylate
RI	refractive index
IR	Infrared
NIR	Near Infrared
RH	Relative humidity
MKR	Microknot resonator
MLR	Microloop resonator
DR	Dynamical Range
PS	Phase shift
